# Nonalcoholic Fatty Liver Disease After Pancreaticoduodenectomy for a Cancer Diagnosis

**DOI:** 10.1089/pancan.2020.0006

**Published:** 2021-03-30

**Authors:** Amy E. McGhee-Jez, Inna Chervoneva, Misung Yi, Amisha Ahuja, Ritu Nahar, Samik Shah, Rebecca Loh, Sarah Houtmann, Rashesh Shah, Charles J. Yeo, Harish Lavu, Steven J. Cohen, Dina Halegoua-DeMarzio, Atrayee Basu Mallick

**Affiliations:** ^1^ Department of Medical Oncology, Thomas Jefferson University Hospital, Philadelphia, Pennsylvania, USA.; ^2^ Division of Biostatistics, Department of Pharmacology and Experimental Therapeutics, Sidney Kimmel Medical College, Thomas Jefferson University, Philadelphia, Pennsylvania, USA.; ^3^ Department of Internal Medicine, Thomas Jefferson University, Philadelphia, Pennsylvania, USA.; ^4^ Department of Surgery, Sidney Kimmel Medical College, Thomas Jefferson University, Philadelphia, Pennsylvania, USA.; ^5^ Division of Hematology/Medical Oncology, Abington/Jefferson Health, Department of Medical Oncology, Thomas Jefferson University Hospital, Willow Grove, Pennsylvania, USA.; ^6^ Division of Gastroenterology and Hepatology, Department of Medicine, Sidney Kimmel Medical College, Thomas Jefferson University, Philadelphia, Pennsylvania, USA.

**Keywords:** NAFLD, Whipple, PPD, risk factors, pancreatic cancer

## Abstract

**Purpose:** Current literature reports increased incidence of postpancreaticoduodenectomy (PD) nonalcoholic fatty liver disease (NAFLD), a precursor for nonalcoholic steatohepatitis and cirrhosis. The incidence of and risk factors (RFs) for NAFLD in the PD population, however, are not well elucidated.

**Methods:** A cohort of 421 patients from a single institution who underwent PD for carcinoma and followed for at least 6 months were assessed retrospectively for age, gender, pathology, surgical complications (operative blood loss and length of stay [LOS]), comorbidities (diabetes, hypertension, hyperlipidemia, obesity), tobacco use, pre- and postoperative nutritional status (albumin and body mass index [BMI]), use of pancreatic enzyme replacement, and perioperative laboratory values (hemoglobin and liver function test). Cox proportional hazards model was used to examine these potential RFs as predictors of time to development of post-PD NAFLD.

**Results:** Sixty (14.3%) patients developed post-PD NAFLD. Patients with NAFLD were younger (61.10 vs. 65.01 years old) and had higher preoperative BMI (28.92 vs. 26.61). Multivariate Cox proportional hazard model identified higher preoperative BMI, shorter postoperative LOS, and female gender as RFs for post-PD NAFLD. After excluding 12 patients with rare histology, there was a lower unadjusted hazard of developing NAFLD (*p*-value = 0.018) in the adenocarcinoma group than in the neuroendocrine and periampullary tumor groups. There was no statistically significant association between post-PD NAFLD and other characteristics.

**Conclusion:** Female gender, higher preoperative BMI, and shorter LOS deserve closer monitoring for earlier detection and management of NAFLD.

## Introduction

Pancreatic cancer is the third-most common cause of cancer-related death in the United States in both men and women.^1^ The incidence was 55,440 in the United States in 2018 with 43,330 patients dying of the disease.^2^ Less than 20% of patients at the time of diagnosis can be offered surgical resection, and thus, those patients undergoing pancreaticoduodenectomy (PD) or distal pancreatectomy represent a relatively small group of patients.^2^ In this population, there is an apparent correlation between patients undergoing PD and the postoperative development of nonalcoholic fatty liver disease (NAFLD).^3–7^ What remains to be investigated, however, is the mechanism by which this develops and factors that put patients at higher risk for its occurrence.

There is limited literature on the study of NAFLD as a consequence of PD, a surgical procedure performed to resect pancreatic masses, including most commonly pancreatic cancer.^8^ PD, commonly referred to as a “Whipple,” includes resection of the pancreatic head, neck, and uncinate process, duodenum, gallbladder, distal common bile duct, and sometimes distal stomach.^8^ NAFLD is an inclusive term encompassing different degrees of severity of fatty liver disease, ranging from steatosis (lipid deposition exceeding 5% of all hepatocytes) to nonalcoholic steatohepatitis (NASH).^4,5^ Steatosis alone is usually a benign finding, however, for those who go onto develop NASH, they are at significant risk (20%) of progression to cirrhosis and potentially hepatocellular carcinoma (HCC), fulminant liver failure, or death.^3^ Currently, NAFLD is one of the most common causes of liver disease worldwide and will likely become the most common in the near future.^9^

A meta-analysis from 2016 investigating the incidence, progression, and overall burden of NAFLD found the estimated worldwide prevalence of NAFLD diagnosed by imaging (ultrasound, computed tomography [CT], and magnetic resonance imaging [MRI]) to be 25% and rising.^10^ Another study found the U.S. incidence to be 25% as well.^11^ NAFLD is quickly becoming a “hepatic manifestation of metabolic syndrome” as it is often seen in association with obesity, diabetes, insulin resistance, hypertension, dyslipidemia, and atherosclerosis.^11^ It is known that NAFLD can progress to NASH, which is one of the leading causes of cirrhosis and HCC, and currently the second leading indication for liver transplant in the United States.^10,11^ The annual incidence of HCC in NAFLD patients, as reported by Younossi et al., was 0.44 per 1000 patient-years and, in patients with NASH, 5.29 per 1000 patient-years.^10^ NAFLD and NASH not only portend a higher risk of liver-related morbidity and mortality, they are also an economic burden in the United States, costing over $100 billion annually for management of the disease and its sequelae.^10,11^

The incidence of NAFLD after PD is reported to be as high as 37% and tends to occur within 12 months of surgery.^3,5^ Few studies have identified consistent risk factors (RFs) for the development of post-PD NAFLD. From the limited literature available, there is suggestion that the risks for post-PD NAFLD differ significantly from those that put the rest of the population at risk for NAFLD, such as obesity and metabolic syndrome.^3,6,7^ It is postulated that exocrine insufficiency and malnourishment are mechanisms of post-PD NAFLD.^3,7^ Kang and Lee suggest that malabsorption of choline, an essential amino acid, can lead to decreased plasma levels of apolipoprotein B and, consequently, impaired hepatic export of triglycerides.^12^ Thus, PD not only alters the anatomy but also the gastrointestinal physiology, which can result in NAFLD.^12^ This deserves further investigation. The aim of the current study was to identify the incidence of and RFs for developing post-PD NAFLD.

## Materials and Methods

Objectives of this study were to identify the overall incidence of NAFLD in patients who have undergone PD for a cancer diagnosis and identify the factors that put these patients at increased risk for developing post-PD NAFLD. We conducted a retrospective chart review of the Thomas Jefferson University Hospital electronic medical record. This study was approved by the Thomas Jefferson University Hospital Institutional Review Board. Patients were identified through a search of the Informatics for Integrating Biology and the Bedside (I2B2) research database available through the TJUH Informatics Department. We identified 769 patients who underwent PD for any cancer diagnosis at our institution from 2007 to 2017. Of these patients, 348 were excluded based on the exclusion criteria listed below. Patients with a diagnosis of pancreatic adenocarcinoma, duodenal adenocarcinoma, ampullary adenocarcinoma, cholangiocarcinoma, pancreatic neuroendocrine tumor, cystic mucinous neoplasm, adenosquamous carcinoma, intraductal oncocytic papillary neoplasm, gastrointestinal stromal tumor, sarcomatoid carcinoma, pancreatic signet ring cell carcinoma, multifocal acinar cell carcinoma, leiomyosarcoma, pancreatic carcinosarcoma, and undifferentiated carcinoma were included. Exclusion criteria included the presence of preoperative hepatic steatosis, PD performed for a noncancer diagnosis (intraductal papillary mucinous neoplasm, pancreatitis, and dysgenic cyst), history of alcohol abuse, history of hepatitis, or <6 months of postoperative follow-up.

The data gathered from these 421 patients meeting the inclusion criteria included age, sex, surgical pathology, surgical complications (including estimated blood loss [EBL] and length of hospital stay), comorbidities (diabetes, hypertension, hyperlipidemia, and obesity), tobacco use, use of postoperative pancreatic enzyme replacement, pre- and postoperative nutritional status (i.e., albumin and body mass index [BMI]), and perioperative laboratory values (hemoglobin and liver function tests). These specific variables were selected based upon review of the literature, including known RFs for non-PD NAFLD and RFs noted to be associated specifically with post-PD NAFLD.^3–6,13–15^ Post-PD NAFLD was identified through review of imaging studies, including abdominal ultrasound, CT, and MRI, as well as clinical progress notes. The time from surgery to identification of NAFLD was recorded.

Baseline characteristics were summarized by means and standard deviations or medians and interquartile ranges for continuous variables, and by counts and percentages for categorical variables by the NAFLD status. The Kaplan–Meier estimator was used to evaluate the time to development of NAFLD in the entire cohort and by the categorical RFs. Univariate Cox proportional hazard model was used to see if a continuous variable had significant impact on the development of NAFLD, and log rank test was used for categorical variables. The Cox proportional hazard model was used for multivariable analysis. For the non-NAFLD group, the observations were considered censored at the time of the last follow-up. The proportional hazard assumption was validated. The following covariates were considered baseline predictors in the Cox model: age (continuous and categorical; <65 and >65 group), EBL (mL), sex, diabetes, hyperlipidemia, hypertension, obesity, tobacco use, length of stay (LOS) (log-transformed number of days in the hospital), as well as preoperative BMI, AST, ALT, alkaline phosphatase, bilirubin, hemoglobin, and albumin. In addition, postsurgery changes in BMI, AST, ALT, alkaline phosphatase, bilirubin, hemoglobin, and albumin were considered time-dependent covariates. The final Cox model was obtained by backward elimination of nonsignificant predictors. Data analysis was performed using SAS 9.4 (SAS Institute, Inc., Cary, NC) and plots were drawn using R 3.5.1 (R Core Team [2014]. R: A language and environment for statistical computing. R Foundation for Statistical Computing, Vienna, Austria. URL www.R-project.org/). The statistical methods of this study were reviewed by Inna Chervoneva, PhD, Associate Professor, Division of Biostatistics, Department of Pharmacology and Experimental Therapeutics at the Sidney Kimmel Medical College of Thomas Jefferson University.

## Results

**FIG. 1.**
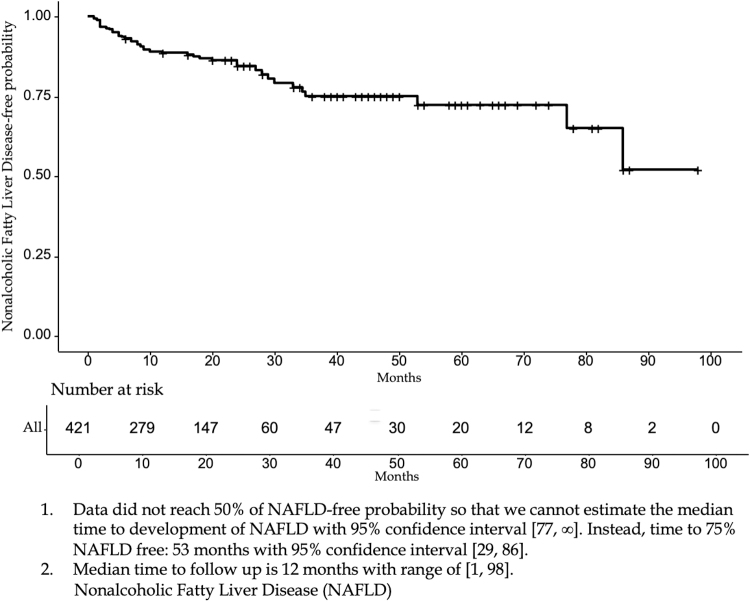
Kaplan–Meier curves of NAFLD-free probability (1). Data did not reach 50% of NAFLD-free probability, and so, we cannot estimate the median time to development of NAFLD with 95% CI (77–∞). Instead, time to 75% NAFLD free: 53 months with 95% CI (29–86). (2) Median time to follow-up is 12 months with range of (1–98). CI, confidence interval; NAFLD, nonalcoholic fatty liver disease.

The study population included 421 patients with at least 6 months of postsurgery follow-up who underwent PD for a cancer diagnosis and did not have evidence of preoperative NAFLD. Table 1 presents baseline characteristics and summary statistics of time to development of fatty liver and use of pancrelipase postoperatively by group for these 421 patients. The median age of patients in the study was 65 years with 187 females and 234 males included. Among them, 60 patients developed fatty liver, making the NAFLD incidence 14.3%. Of those who developed post-PD NAFLD, 32/60 (53.3%) were female and 28/60 (46.6%) were male. Baseline characteristics of our population showed patients with NAFLD were younger (61.10 vs. 65.01 years old) and had higher preoperative BMI (28.92 vs. 26.61). In univariate analysis, only shorter LOS had a significant effect on the hazard of developing post-PD NAFLD (*p* = 0.029). There was no difference in use of postoperative pancrelipase between the two groups (91.67% and 95.52%, respectively; *p* = 0.065). Our data did not reach 50% of NAFLD-free survival, so we were unable to report the median time to development of NAFLD with a 95% confidence interval. Alternatively, the time to 75% NAFLD-free survival was 53 months (95% CI: 29, 86; Fig. 1).

**Table 1.** Baseline Characteristics and Univariate Analysis for Risk of Postpancreaticoduodenectomy Nonalcoholic Fatty Liver Disease

	All (n = 421)	NAFLD (n = 60)	No NAFLD (n = 361)	p^a^
Median [IQR] or mean (SD)				
Age (years)	65 [56–72]	62 [53–68]	65 [57–73]	0.030^b^
Log_2_ LOS (days)	2.8 [2.6–3.2]	2.8 [2.6–3.0]	2.8 [2.6–3.3]	0.029^b^
BMI preoperatively (kg/m^2^)	26.9 (5.0)	28.9 (6.0)	26.6 (4.8)	0.002^b^
EBL preoperatively (mL)	474.4 (493.7)	515.4 (391.6)	466.4 (511.5)	0.485
AST preoperatively	73.7 (99.2)	51.6 (43.8)	77.5 (105.4)	0.108
ALT preoperatively	105.5 (145.7)	77.6 (100.8)	110.2 (151.5)	0.159
AP preoperatively	238.6 (252.2)	214.5 (402.8)	242.6 (217.7)	0.598
Bilirubin preoperatively	3.0 (4.6)	3.0 (4.6)	3.0 (4.6)	0.893
Hb preoperatively	12.2 (1.7)	12.3 (1.6)	12.2 (1.7)	0.735
Albumin preoperatively	3.9 (0.6)	4.0 (0.6)	3.8 (0.6)	0.132
Sex, *n* (%)				0.092
Female	187 (44)	32 (53)	155 (43)	
Male	234 (56)	28 (47)	206 (57)	
Age, *n* (%)				0.111
<65	224 (53)	38 (63)	186 (52)	
>65	197 (47)	22 (37)	175 (48)	
BMI preoperatively, *n* (%)				0.341
Underweight (<18.5)	59 (14)	7 (12)	52 (14)	
Normal (18.5–25)	145 (34)	15 (25)	130 (36)	
Overweight (25–30)	129 (31)	21 (35)	108 (30)	
Obesity (>30)	88 (21)	17 (28)	71 (20)	
Diabetes, *n* (%)				0.990
Yes	118 (28)	18 (30)	100 (28)	
No	303 (72)	42 (70)	261 (72)	
HLD, *n* (%)				0.382
Yes	168 (40)	28 (47)	140 (39)	
No	253 (60)	32 (53)	221 (61)	
HTN, *n* (%)				0.210
Yes	240 (57)	39 (65)	201 (56)	
No	180 (43)	21 (35)	159 (44)	
Obesity, *n* (%)				0.485
Yes	99 (24)	17 (28)	82 (23)	
No	315 (76)	43 (72)	272 (77)	
Tobacco use, *n* (%)				0.307
Yes	197 (47)	31 (52)	166 (46)	
No	224 (53)	29 (48)	195 (54)	
Use of pancrelipase postoperatively, *n* (%)				0.065
Yes	396 (95)	55 (92)	341 (96)	
No	21 (5)	5 (8)	16 (4)	

^a^ Univariate Cox proportional hazard model was used to see if a continuous variable has a significant impact on the development of NAFLD, and log-rank test was used for categorical variables.

^b^ *p* < 0.05.

ALT, alanine aminotransferase; AP, alkaline phosphatase; AST, aspartate aminotransferase; BMI, body mass index; EBL, estimated blood loss; Hb, hemoglobin; HLD, hyperlipidemia; HTN, hypertension; IQR, interquartile range; LOS, length of stay; SD, standard deviation.

**FIG. 2.**
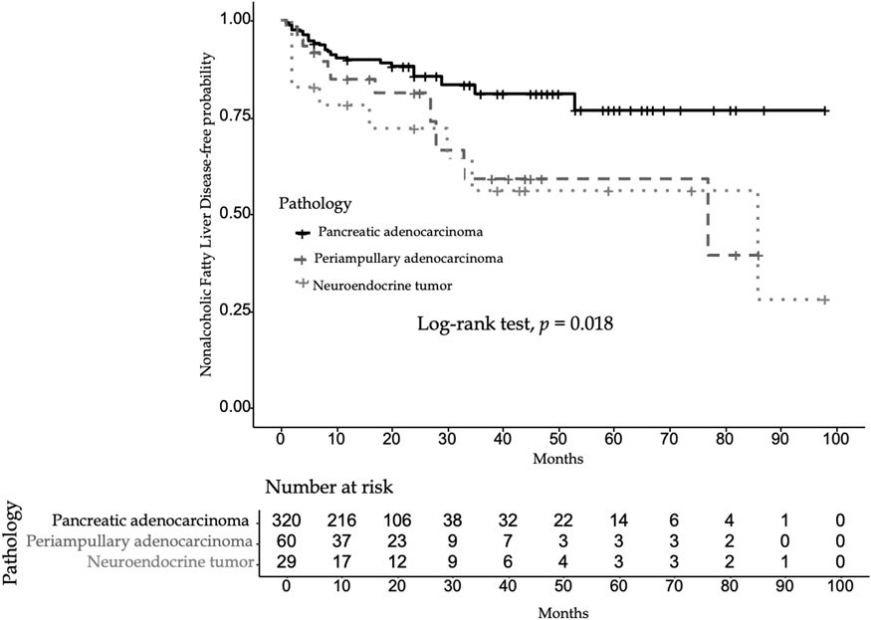
Kaplan–Meier curve of NAFLD probability by tumor type. This displays lower unadjusted hazard of developing fatty liver in the pancreatic adenocarcinoma group than in the neuroendocrine and periampullary tumor groups.

Distribution of tumor types among patients with and without post-PD NAFLD was also examined. Tumor types were divided as follows: pancreatic adenocarcinoma, pancreatic neuroendocrine tumors, and periampullary adenocarcinomas (including ampullary adenocarcinomas, duodenal adenocarcinomas, and cholangiocarcinomas), all requiring PD. Twelve patients with rare histology were excluded from this analysis, given the small number of patients. There was a significant effect of tumor type on the unadjusted hazard of developing fatty liver (*p*-value = 0.018). The Kaplan–Meier curve of NAFLD-free probability by tumor types in Figure 2demonstrates lower unadjusted hazard of developing fatty liver in the pancreatic adenocarcinoma group than in the neuroendocrine and periampullary tumor groups.

The results of the multivariate Cox proportional hazard model (Table 2) demonstrated that sex, preoperative BMI, and hospital LOS had significant effect on the hazard of developing NAFLD. Females had 86.3% higher hazard of development of NAFLD compared with males (hazard ratio [HR] = 1.863, 95% CI: 1.068, 3.251; *p* = 0.028). One-unit increase in BMI implied an 11.2% increase in the hazard of NAFLD development (HR = 1.112, 95% CI: 1.004, 1.232; *p* = 0.041). Moreover, a doubling of postoperative hospital LOS was associated with a 55% decrease in the hazard of developing NAFLD (HR = 0.448, 95% CI: 0.217–0.922; *p* = 0.029). The remainder of the RFs considered were not statistically significant predictors of NAFLD development (Table 3).

**Table 2.** Results from Final Multivariate Cox Proportional Hazard Model, Including Shorter Postoperative Hospital Stay, Female Sex, and Higher Preoperative Body Mass Index

Effect	HR	95% HR CI		p
Log_2_ (LOS)	0.45	0.22	0.92	0.029
Sex, female	1.86	1.07	3.25	0.028
BMI (preoperative)	1.11	1.00	1.23	0.041

CI, confidence interval; HR, hazard ratio.

**Table 3.** List of Multivariate Analyses Performed with Results That Were not Statistically Significant

No.	Models
1	Models with age (continuous and categorical; <65 and >65 group), EBL, diabetes, HLD, HTN, obesity, tobacco use, or alcohol abuse.
2	Models with LOS.
3	Model with time-dependent variable, BMI (1 month, 6 months, 1 year, 2 years, and 3 years postoperative).
4	Model with time-dependent variable, albumin (1 month, 6 months, 1 year, 2 years, and 3 years postoperative).
5	Models with tumor types.

## Discussion

Our study is the largest single-institution study examining RFs for post-PD NAFLD performed for a cancer diagnosis. We found the incidence of post-PD NAFLD to be 14.3%, consistent with that seen in other reports.^3–6,16^ In addition, we identified female sex, higher preoperative BMI, and shorter LOS as potential RFs for development of post-PD NAFLD. Our study adds to the literature with regard to identifying RFs for post-PD NAFLD and confirming this association. It is important to report this information so that physicians may better educate patients on their risk and investigate interventions to avoid development or progression of NAFLD.

Previous studies of this patient population have noted a difference in the RFs and patient characteristics associated with post-PD NAFLD/NASH, compared with patients who developed these diagnoses without having undergone PD.^3^ In particular, they have noted that the typical non-PD patient population with NAFLD/NASH is often obese and has comorbidities, including diabetes, hyperlipidemia, and insulin resistance.^3,16^ In the patients having undergone PD, on the contrary, they were less likely to be obese or have these comorbidities.^3,6,7^ Interestingly, they tended to be malnourished with lower BMI, albumin, and fasting insulin concentrations postoperatively.^3,6,7^ Many authors hypothesize that this is related to pancreatic exocrine insufficiency and malabsorption, but the exact mechanism has not yet been elucidated.^7,12^

Proposed RFs for the development of post-PD NAFLD have included pancreatic head cancer, gender, age >70 years old, certain laboratory abnormalities (high postoperative transaminases, for example), surgical blood loss, pancreatic texture, pancreatic duct fistula, external pancreatic duct stenting, and poor postoperative nutritional status.^3–6,16^ These identified RFs were not consistent across all studies, which is why our study contributes to this growing literature.

Our study, like the study by Ivanics et al., also demonstrated that female sex is an RF for post-PD fatty liver on multivariate analysis.^4^ Ivanics et al. reported that 61% (19/31) of the patients who developed NAFLD after pancreatic resection (PD, distal pancreatectomy, or total pancreatectomy) in their study were female, which was statistically significant (*p* = 0.05).^4^ On the contrary, Olefson et al. found a significant interaction of liver to muscle ratio (LMR) with sex.^6^ They used LMR as a surrogate for fat deposition in the liver and found a significant reduction in pre- and postoperative LMR only in men from 1.81 (95% CI [1.63–1.93]) to 1.50 (95% CI [1.37–1.63]; *p* = 0.003).^6^ Thus, our study supports the conclusions of Ivanics et al. in female sex as an RF.

Unlike other studies, however, we were not able to demonstrate that malnourishment, represented by lower preoperative BMI or albumin, or a change in BMI and albumin postoperatively, was an RF for post-PD NAFLD. On the contrary, multivariable analysis showed that a higher preoperative BMI in this patient population had a significant effect on the risk of developing post-PD NAFLD.

In addition, our finding that patients with a shorter postoperative hospital stay had a higher hazard of developing post-PD NAFLD appears counterintuitive. It may suggest that patients with better nutritional status before surgery, and thus a higher preoperative BMI, had a higher hazard of developing fatty liver, although we do not have evidence to support this. Another possible explanation relates to the pancreatic remnant texture. Beginning in 2015, Lavu and colleagues at our institution studied and subsequently demonstrated the feasibility and safety of a postoperative day (POD) 5 discharge after PD versus a POD 7 discharge in patients with hard pancreatic remnants.^17^ Consequently, it is possible that this patient population with hard pancreatic remnants, who can better tolerate an earlier hospital discharge, has a higher hazard of developing post-PD NAFLD. Our study, however, did not specifically investigate pancreatic remnant texture and so this remains a hypothesis. Last, it is possible that those patients who are discharged earlier are more fit and thus able to receive earlier and more adjuvant chemotherapy. The chemotherapy may, in fact, be the RF for post-PD NAFLD in this case. Unfortunately, we were unable to study this variable in our patient population. No other preoperative or postoperative RFs identified in the multivariable analysis were found to be statistically significant.

Interestingly, when looking for effect of tumor type on the development of fatty liver, we identified patients with pancreatic neuroendocrine tumors and periampullary adenocarcinomas to be at higher risk than those patients with pancreatic adenocarcinoma, although this did not reach statistical significance on multivariate analysis. This may be related to longer life expectancy and thus more time to develop fatty liver in this patient population compared with patients with pancreatic adenocarcinoma. It could also be explained by the overall small number of patients who had pancreatic neuroendocrine and periampullary tumors in this study (89 total, 23 of whom developed post-PD NAFLD).

Furthermore, proposed therapeutic interventions in patients who develop post-PD NAFLD have included close clinical and laboratory surveillance after surgery, aggressive nutritional assessment, and pancreatic enzyme repletion in the postoperative setting.^4,6,7,14^ Some studies even reported gradual improvement in steatosis after these interventions.^3,7,13^ Our study, however, found no statistically significant difference between those patients who did and did not develop NAFLD in the use of postoperative pancreatic enzyme repletion (91.67% and 94.52%, respectively; *p* = 0.199).

A limitation of our study included the relatively small number of patients who developed post-PD NAFLD (60), despite this being the largest single-institution study examining RFs for post-PD NAFLD performed for a cancer diagnosis. In addition, like many other studies, we were unable to study the effect of chemotherapy on the development of NAFLD as a large number of patients did not receive their neoadjuvant or adjuvant therapy at our institution and outside records were not available. The only study to date that has directly investigated a possible association between post-PD NAFLD and adjuvant chemotherapy is a retrospective study of 154 patients from Japan, which showed a significant increase in the incidence of post-PD NAFLD from 19% to 38% with the addition of adjuvant chemotherapy, specifically with an adjuvant S-1 regimen (tegafur/gimeracil/oteracil potassium).^18^

While our current study is also a retrospective chart review, it has a number of strengths. It is a single-institution study, in which there is a small population of pancreatic surgeons and medical oncologists who follow strict protocol with regard to surgical procedure and postoperative management of patients undergoing PD. Consequently, this is a homogenous patient population that was studied. While we did not have chemotherapy data available, we were able to study the most known and predicted RFs for the development of post-PD NAFLD. This study is hypothesis generating and supports the need for prospective data. This could be achieved by adding surveillance for post-PD NAFLD to those patients enrolled in clinical trials. Adjuvant therapy is improving, as evidenced by the PRODIGE 24 trial, where the median overall survival was extended to 54.4 months from 35 months with the use of adjuvant FOLFIRINOX versus gemcitabine monotherapy.^19^ Consequently, NAFLD will likely become a survivorship issue that physicians will have to manage more frequently in the future.

## Conclusion

In conclusion, this retrospective chart review confirms that there is an increased incidence of NAFLD after PD performed for a cancer diagnosis and patients who are of female sex, have shorter postoperative hospital stays, or have higher preoperative BMI may be at increased risk for its development. These patients may require closer monitoring postoperatively to achieve earlier detection and earlier intervention in the form of enzyme replacement and aggressive nutritional assessment to prevent progression to overt NASH or cirrhosis.

## Abbreviations Used

ALT: alanine aminotransferase
AP: alkaline phosphatase
AST: aspartate aminotransferase
BMI: body mass index
CI: confidence interval
CT: computed tomography
EBL: estimated blood loss
Hb: hemoglobin
HCC: hepatocellular carcinoma
HLD: hyperlipidemia
HR: hazard ratio
HTN: hypertension
I2B2: Informatics for Integrating Biology and the Bedside
IQR: interquartile range
LMR: liver to muscle ratio
LOS: length of stay
MRI: magnetic resonance imaging
NAFLD: nonalcoholic fatty liver disease
NASH: nonalcoholic steatohepatitis
POD: postoperative day
RF: risk factor
SD: standard deviation
